# Oil-based versus water-based contrast media for hysterosalpingography in infertile women of advanced age, with ovulation disorders or a high risk for tubal pathology: study protocol of a randomized controlled trial (H2Oil2 study)

**DOI:** 10.1186/s12905-022-01707-z

**Published:** 2022-04-18

**Authors:** K. Rosielle, D. Kamphuis, N. van Welie, I. Roest, A. Mozes, E. J. P. van Santbrink, T. van de Laar, A. B. Hooker, A. G. Huppelschoten, W. Li, M. Y. Bongers, J. Stoker, M. van Wely, C. Koks, C. B. Lambalk, A. Hemingway, B. W. J. Mol, K. Dreyer, V. Mijatovic

**Affiliations:** 1 Department of Reproductive Medicine, Amsterdam UMC location Vrije Universiteit Amsterdam, De Boelelaan 1117, 1081 HV Amsterdam, The Netherlands; 2 Amsterdam Reproduction and Development, Amsterdam, The Netherlands; 3 Grow Research School for Oncology and Reproduction, Maastricht University, Universiteitssingel 40, 6229 ER Maastricht, The Netherlands; 4 Department of Obstetrics and Gynaecology, Máxima MC, De Run 4600, 5504 DB Veldhoven, Eindhoven, The Netherlands; 5 Department of Obstetrics and Gynaecology, Ziekenhuis Amstelland, Laan van de Helende Meesters 8, 1186 AM Amstelveen, The Netherlands; 6 Department of Reproductive Medicine, Reinier de Graaf Gasthuis, Reinier de Graafweg 5, 2625 AD Delft, The Netherlands; 7 Department of Obstetrics and Gynaecology, Elkerliek Ziekenhuis, Wesselmanlaan 25, 5707 HA Helmond, The Netherlands; 8 Department of Obstetrics and Gynaecology, Zaans Medisch Centrum, Koningin Julianaplein 58, 1502 DV Zaandam, The Netherlands; 9 Department of Obstetrics and Gynaecology, Catharina Ziekenhuis, Michelangelolaan 2, 5623 EJ Eindhoven, The Netherlands; 10 Department of Obstetrics and Gynaecology, Monash University, 246 Clayton Rd, Clayton, VIC 3168 Australia; 11 Department of Radiology and Nuclear Medicine, Amsterdam UMC location University of Amsterdam, Meibergdreef 9, 1105 AZ Amsterdam, The Netherlands; 12 Centre for Reproductive Medicine, Amsterdam Reproduction and Development, Amsterdam UMC location University of Amsterdam, Meibergdreef 9, 1105 AZ Amsterdam, The Netherlands; 13 Department of Radiology, Imperial College Healthcare NHS Trust, Hammersmith Hospital, DuCane Road, London, W12 0HS England

**Keywords:** Hysterosalpingography (HSG), Tubal patency testing, Cost–benefit analysis, Ethiodized oil, Infertility, Ongoing pregnancy, Oil-based contrast, Water-based contrast

## Abstract

**Background:**

In women with unexplained infertility, tubal flushing with oil-based contrast during hysterosalpingography (HSG) increases ongoing pregnancy and subsequent live birth rates when compared to tubal flushing with water-based contrast. It is currently unclear whether an HSG with oil-based contrast also results in more ongoing pregnancies and live births in women of advanced age, women with ovulation disorders, and women with potential tubal pathology when compared to an HSG with water-based contrast.

**Methods:**

We plan an international, multicentre, open-label, randomized controlled trial (RCT) studying three groups of infertile women who have an indication for tubal patency testing according to their treating physician and additionally; (1) are 39 years of age or older, (2) have an ovulation disorder or (3) have a high risk for tubal pathology based on their medical history. Women with an allergy for iodinated contrast medium are excluded, as are women with diabetes, hyperprolactinemia or untreated hyper- or hypothyroidism, and women with a partner with severe male infertility. After informed consent, women will be randomly allocated to the intervention, tubal flushing with the use of oil-based contrast during HSG or the control group, tubal flushing with the use of water-based contrast during HSG in a 1:1 ratio by the web-based system Castor. The primary endpoint will be ongoing pregnancy leading to live birth with conception within six months after randomization. Secondary outcomes are other pregnancy outcomes, used fertility treatments, adverse events and cost-effectiveness. Based on the expected ongoing pregnancy rate of 17% in the control group and 27% in the intervention group, the sample size will be 930 women (465 per group). Study inclusion is expected to be complete in four years.

**Discussion:**

This multicentre RCT will establish whether, for women of advanced age, women with ovulatory disease, and women who have a high risk for tubal pathology, there is a fertility enhancing effect of tubal flushing with oil-based contrast during HSG and whether the use of this contrast medium is cost-effective.

*Trial Registration* The study was prospectively registered in the Netherlands Trial Register on August 1st 2019 as ‘H2Oil2’ (reference number NL7925, https://www.trialregister.nl/trial/7925).

**Supplementary Information:**

The online version contains supplementary material available at 10.1186/s12905-022-01707-z.

## Background

Infertility, defined as the inability to conceive within 12 months of unprotected intercourse, was estimated to effect approximately 48.5 million couples worldwide in 2010 [1, 2]. Fertility work-up includes the medical investigation into the various causes of male and female infertility that have been identified. With male factor infertility referring to the (relative) absence of functioning spermatozoa, female infertility can refer to ovulation disorders, diminished ovarian reserve, tubal factor infertility, and uterine factor infertility [3]. Ovulation disorders can have various causes, of which polycystic ovary syndrome (PCOS) is the most prevalent [4]. Diminished ovarian reserve plays an increasing role in infertility as couples have been postponing their wish to conceive over recent decades [5]. Tubal factor infertility can be caused by current or past pelvic inflammatory disease such as an infection with *Chlamydia trachomatis*, previous pelvic surgery, peritonitis or endometriosis [6, 7]. Uterine factor infertility can consist of anatomical anomalies, such as congenital uterine anomalies, or intrauterine pathology such as polyps, myomas or adhesions [8]. In up to 30% of couples, the fertility work-up shows no abnormalities and this is classified as unexplained infertility [9, 10].

Part of the fertility work-up is assessing the risk for tubal pathology and if indicated, a tubal patency test can be performed. Hysterosalpingography (HSG) is traditionally used as first choice tubal patency test to rule out tubal pathology [3, 4]. Although HSG was initially introduced as a diagnostic test, therapeutic effects of tubal flushing, especially with oil-based contrast, have been studied extensively [11–14]. The most recent review [14] included six studies comparing HSG with oil-based contrast and water-based contrast. Three of these studies reported on live birth but meta-analysis could not be performed due to heterogeneity of the population.

The largest study among these randomized controlled trials is the H2Oil study [15]. This study was conducted to investigate the difference in ongoing pregnancy rates between tubal flushing during HSG with the use of oil-based and water-based contrast, in couples with unexplained or mild male infertility. This study excluded women aged 39 years or older, women with ovulation disorders, and women who had a high risk for tubal pathology. The H2Oil study showed a significant increase in ongoing pregnancies as well as live births within 6 months after HSG with oil-based contrast when compared to HSG with water-based contrast (relative risk (RR) 1.37, 95% confidence interval (CI) 1.16–1.61; P < 0.001 for ongoing pregnancy and RR 1.38, 95% CI 1.17 to 1.64; P < 0.001 for live birth) [15]. The long term follow-up of this study demonstrated that the fertility enhancing effect of oil-based contrast is still present five years after HSG (cumulative ongoing pregnancy rates 80.0% after oil-based contrast, 75.0% after water-based contrast, RR 1.07, 95% CI 1.00 to 1.14, cumulative live birth rates 74.8% after oil-based contrast, 67.3% after water-based contrast, RR 1.11; 95% CI 1.03 to 1.20) [16]. This study additionally demonstrated that HSG with oil-based contrast leads to a significantly shorter time-to-pregnancy compared to HSG with water-based contrast (10.0 vs 13.7 months; hazard ratio 1.25; 95% CI 10.9 to 1.43). Robust studies investigating the fertility enhancing effect of oil-based contrast during HSG in women who were 39 years of age or older, women with ovulation disorders, and women who have a high risk for tubal pathology are lacking.

In the current socio-economic climate, where health care costs are increasing and the importance of evidence-based health care is underlined, the results of previously mentioned studies among couples with unexplained infertility cannot be extrapolated to couples with other types of infertility and therefore separate evaluation is needed.

This randomized controlled trial aims to investigate the effectiveness and cost-effectiveness of the use of oil-based versus water-based contrast during HSG, in women with previously unevaluated causes of infertility: women who are 39 years of age or older, women with ovulation disorders, and women who have a high risk for tubal pathology.

## Methods

This international, multicentre, randomized controlled trial will be performed in university, teaching and non-teaching hospitals in the Netherlands and the United Kingdom. A list of currently participating hospitals is available as Additional file 2*.* The trial has obtained ethical approval by the Institutional Review Board (IRB) of the Amsterdam UMC location Vrije Universiteit (registration number 2018.289), the Research Ethics Committee London Harrow (20/LO/0607), and the board of directors of all participating centres.

### Participants

Women who are scheduled for an HSG as part of their fertility work-up can participate if they meet at least one of the following criteria: (1) women who are 39 years of age or older, (2) women who have an ovulation disorder (ovulation disorders will be defined as less than eight menstrual cycles per year), or (3) have a high risk for tubal pathology (high risk will be defined as a past chlamydia infection, pelvic inflammatory disease, peritonitis, known endometriosis and/or pelvic surgery including tubectomy for ectopic pregnancy). In order to ensure adequate sample sizes in all three groups of participants, women meeting more than one criterion will be included according to the criterion that comes first in ranking. The ranking is based on the expected prevalence of the three subgroups of women within the study population, with the lowest expected prevalence highest in ranking. Women will be excluded if they have an endocrine disorder known to decrease natural pregnancy chances (e.g. diabetes, unregulated hypothyroidism or hyperthyroidism), iodine contrast medium allergy or a male partner with severe infertility (a pre-washed total motile sperm count below three million sperm per millilitre).

### Randomization and blinding

Infertile couples will be screened in the outpatient clinic by their attending physician. Eligible women will be informed by a dedicated research nurse or physician in their centre. Women who give written consent will be randomized for HSG with oil-based contrast (intervention group) or with water-based contrast (control group) in a 1:1 ratio, using a permuted block design with block sizes varying from 4–8 cases. Randomization will be performed using the web-based program Castor EDC (Castor Electronic Data Capture, Ciwit BV, Amsterdam, the Netherlands), and stratified according to centres and by infertility diagnosis (age 39 years or older, ovulation disorder, and high risk for tubal pathology). The trial is not blinded with respect to participants and health care professionals since the allocation will be evident in further fertility workup. Oil-based contrast has a higher iodine concentration than water-based contrast and together with its hydrophobic qualities this makes oil-based contrast easily distinguishable from water-based contrast on X-ray or fluoroscopy images [17]. The primary outcome is objective, and therefore we do not expect that lack of blinding will influence the findings.

### Intervention

The HSG procedure will be performed after cessation of menstrual bleeding or after progesterone-induced vaginal bleeding in case of anovulation. With use of a cervical vacuum cup, a metal cannula (hysterophore), an acorn cannula or an HSG balloon catheter, the iodinated oil- or water-based contrast medium will be infused into the uterine cavity and several radiographs will be taken to visualize the uterine cavity and Fallopian tubes according to local protocols. The procedure will be discontinued if signs of intravasation are visible on the radiographs, as intravasation of specifically oil-based contrast might lead to oil-embolisms, a known and potentially dangerous complication [18]. The results of the HSG will describe whether the Fallopian tubes are patent and whether there are any visual abnormalities in the cervix, uterine cavity, Fallopian tubes or peritoneal cavity. In the intervention arm, the HSG will be performed with a maximum of 15 millilitre of oil-based contrast (Lipiodol Ultra Fluid®, Guerbet, Villepinte, France) to minimize the chance of (temporary) hypo- or hyperthyroidism [18]. In the comparator arm, the HSG will be performed with water-based contrast medium (iodixanol, Visipaque®, General Electric Healthcare, Buc, France), for which no maximum dosage is advised. The batch number and expiration date of the used flasks of contrast medium will be reported for the purpose of drug accountability.

Pre- and post-HSG use of analgesics and antibiotics, and subsequent management will be performed according to local protocol. Women will receive the adjusted Amsterdam Preoperative Anxiety and Information Scale (APAIS) questionnaire prior to their HSG procedure to score their anxiety score prior to HSG and to be able to relate this to their pain level during HSG [19]. Immediately after the procedure they will be asked to score their pain using a Visual Analogous Scale (VAS) ruler (ranging from 0 to 10 cm).

The choice of fertility treatments will be based on the results of the fertility work-up (including the outcome of the HSG) according to the Dutch fertility Guideline or the Clinical guideline by the National Institute for Healthcare and Excellence (NICE) [4, 20–22]. Anovulatory women will be offered ovulation induction, and women aged 39 years or older may be offered Intra Uterine Insemination (IUI) or In Vitro Fertilization (IVF). In case of suspected uni- or bilateral tubal pathology, women can be scheduled for IVF or a diagnostic or therapeutic laparoscopy followed by IVF if bilateral tubal occlusion is confirmed, according to the local protocol of the participating centres. Women with a high risk for tubal pathology, but without tubal pathology at HSG or laparoscopy, and with a regular menstrual cycle who are below 39 years of age will be advised expectant management or IUI, guided by their calculated prognosis for natural conception using the model of Hunault or other local protocols [23]. For women aged 39 or over, the Hunault prognostic model is not verified and these women will often be advised IUI or IVF immediately.

As the compared strategies (HSG with oil-based contrast versus HSG with water-based contrast) are already applied in current practice, no additional risks or burdens are expected for participating women.

### Outcomes

The primary outcome of this trial is ongoing pregnancy leading to live birth, with conception within six months after randomization. Ongoing pregnancy will be defined as an intrauterine pregnancy with heartbeat on ultrasound examination at twelve weeks of gestation, live birth as a live born neonate beyond 24 weeks of gestation. Secondary outcomes will include clinical pregnancy (ultrasound confirmed intrauterine gestational sac), ongoing pregnancy, miscarriage (loss of clinical or ongoing pregnancy or diagnosis of a pregnancy without positive foetal heartbeat before twelve weeks gestation), ectopic pregnancy (ultrasound or surgically confirmed extra-uterine pregnancy). Pregnancy complications, complications of HSG such as intravasation, infection and hypo- or hyperthyroidism, and a cost-effectiveness analysis will also be part of the secondary outcomes. Our hypothesis is that HSG with oil-based contrast will increase ongoing pregnancy rates and will reduce time to ongoing pregnancy in all three subgroups, thereby reducing the need for assisted reproductive technology (ART) and thus lowering the costs. In addition, we will study the procedural discomfort or pain and relate this to pre-procedural anxiety, using a modified APAIS questionnaire [19].

### Follow-up

Data on fertility treatments and pregnancy outcomes will be collected until six months after randomization in a structured electronic case report form using Castor EDC. If a pregnancy occurs within six months, the outcome of the pregnancy will be followed. If the necessary information cannot be extracted from the medical record, women will receive a digital questionnaire about treatment and pregnancy outcomes or they will be contacted by a dedicated researcher to conduct the follow-up questionnaire by phone. All participating women will receive a digital questionnaire on productivity-loss and health care costs (iPCQ) six months after randomization (see Table 1) [24].

**Table 1:** SPIRIT figure

Time-point	Study period				
	Enrollment	Allocation	Post-allocation		
	t−1	t0	t1	t2	t3
Enrollment					
Eligibility screen	X				
Informed consent	X				
Allocation		X			
Interventions					
HSG with oil-based contrast			X		
HSG with water-based contrast			X		
Assessments					
Demographics		X			
APAIS			X		
Pain score (VAS)			X		
HSG procedure and results			X		
Adverse effects			X		
iPCQ				X	
Treatments				X	
Pregnancies				X	
Pregnancy follow-up					X

t−1: Prior to inclusion; t0: Study inclusion; t1: HSG procedure, usually within 4 weeks of inclusion (t0); t2: end of initial follow-up 6 months after randomization; t3: pregnancy follow-up, at last 9 months after t2

### Sample size

Our hypothesis is that HSG with oil-based contrast will increase the live birth rate with 10% in three infertility groups: (1) women aged 39 years or older, (2) women with ovulation disorders and (3) women who have a high risk for tubal pathology. To detect an increase of 10% in live birth rate from 17 to 27%, 395 women per group are needed (alpha 1%, beta 20%, Z-test with unpooled variances as calculated in PASS 2020). Anticipating a loss to follow-up rate of 15%, the total number of participants required is 930 (465 in each arm of the trial). With this number we have 80% power to study the 10% difference in live birth rate in the intervention versus control group in the stratified design using Cochrane-Mantel-Haenzel.

### Statistical analysis

Categorical data will be reported as absolute numbers and percentages. Normally distributed continuous variables will be summarized as means with standard deviations, and non-normally distributed continuous variables will be reported as medians with interquartile ranges. The primary analyses will done according to the intention to treat (ITT) principle, including all randomised women. Differences in live births will be expressed as crude and stratification adjusted risk ratio and absolute risk difference with associated 95% and 99% confidence intervals (CI) using log-linear binomial regression. We will construct Kaplan–Meier curves, estimating the cumulative probability of conception leading to live birth over time and use the log-rank test to assess differences. Additionally, we will do a cox proportional hazard analysis to evaluate the difference in primary outcomes over time while accounting for the subgroups and evaluating presence of interaction. Continuous outcomes will be measured at multiple time-points and will be analysed with the use of linear mixed models. We will subsequently compare intervention and control treatment within the stratified groups (1) women aged 39 years or older, (2) women with ovulation disorders and (3) women who have a high risk for tubal pathology. Women meeting more than one criterion will be included in the group that comes first in ranking as described earlier. Within these stratified groups pregnancy outcomes will be expressed as risk ratio, risk difference and hazard ratio with 95% CI.

### Cost-effectiveness analysis

The average costs and effects of tubal flushing during HSG with oil-based contrast and water-based contrast during fertility work-up will be compared. Total costs of the HSG, fertility treatments and fertility outcomes (collected using the eCRF) will be evaluated after a follow-up of six months after randomization. These data will be used to calculate the direct medical costs.

Societal costs will be measured using digital iPCQ questionnaires after six months of follow-up [24]. Cost categories that will be included are: (1) healthcare costs (primary and secondary care, complementary care and home care); (2) lost productivity costs (absenteeism from paid and unpaid work, and presentism) and (3) patient costs (informal care and other care services paid for by patients themselves).

Valuation for participants from the Netherlands and the United Kingdom will be according to their respective national guidelines [25, 26]. For the valuation of health care utilization, lost productivity and informal care, standard costs for the Netherlands and the United Kingdom will be used. Medication use will be valued using prices of the Royal Dutch Society for Pharmacy (www.KNMP.nl) and the NICE British National Formulary (www.bnf.nice.org.uk). Patient and family costs other than informal care will be valued using self-reported prices. For the valuation of absenteeism from paid work, the friction cost approach will be used.

### Safety monitoring

The IRB determined that the study related risk for participants is very low. A Data Safety Monitoring Committee was therefore not deemed necessary. An interim analysis is not planned. All adverse events (AEs) occurring within one month after HSG will be reported to the IRB by line listing yearly. Additionally, adverse neonatal outcomes such as a congenital anomaly or birth defect will be reported as severe adverse events (SAEs). SAEs occurring within one month after HSG will be reported to the IRB immediately through the Dutch national web portal ToetsingOnline. SAEs occurring in participants outside of the Netherlands will additionally be processed according to local regulations. All SAEs will be followed until they have abated, until a stable situation has been reached or the patient was discharged.

### Data management and monitoring

Patient information will be filled in anonymously based on randomization number. Linking personal data to the study number can only be performed in the local participating centres. Written informed consent forms are stored in the local participating centre, all forms and data will be archived for 25 years in the participating centres according to GCP and local regulations. Monitoring of study processes will be done according to national and international guidelines by an independent study monitor [27]. Annual safety reports will be sent to the accredited IRB and the competent authority.

See Additional file 1: Table S1 for the completed WHO Trial Registration Data Set [28].

## Discussion

Increasing female age is one of the main causes of infertility in the twenty-first century, with > 50% of the women undergoing IVF being over 35 years of age [29, 30]. Anovulation and tubal pathology are also important causes of infertility. As a consequence, the results of this H2Oil2 study are estimated to be applicable to more than 50% of infertile women seen in fertility clinics.

This multicentre randomized controlled trial will generate insight in the potential fertility enhancing effect of tubal flushing using oil-based contrast during HSG in infertile women who are 39 years or older, women with ovulatory disease, and women with a high risk for tubal pathology. The generated evidence can guide clinicians and policy makers to decide which subgroups of women will benefit from an HSG with oil-based contrast as a therapeutic intervention, whether the intervention is cost-effective and if the risk of adverse events is acceptable.

### Strengths and limitations

The proposed study is specifically designed to detect a difference in ongoing pregnancies leading to live births for three important subgroups of infertile women. Most randomized clinical trials regarding the fertility enhancing effect of oil-based contrast during HSG have been previously performed in couples with unexplained infertility and/or mild male factor. While several trials have also included women with other types of infertility [31–34], none were able to show a significant positive effect of tubal flushing with oil-based contrast mostly due to a low sample size, and the results of previous trials among women with unexplained infertility cannot simply be extrapolated to all women facing infertility [14]. Another strength of this study is its multinational character. As it involves academic and non-academic (teaching and non-teaching) participating centres from the Netherlands and the United Kingdom, the results will be applicable to different countries with different hospital settings. A limitation of this study is the potential diversity in treatments between the various participating centres. The participating centres will treat patients according to their local protocol. Although these protocols are based on national guidelines, there is variety between the national guidelines of the participating countries [4, 35]. As this study evaluates two variants of standard care which are already applied in current practice, we chose this pragmatic approach to generate evidence that is applicable to the majority of the treating centres. This approach will lead to a difference in management between participating centres, possibly influencing the chance of conception. Randomization is therefore stratified per inclusion group and per inclusion site, to prevent uneven distribution among the two randomization groups.

### Potential impact and implications

As health care costs are increasing around the world, research focusing on cost-effectiveness of healthcare will help clinicians and policy makers to determine the appropriate position of the HSG with oil-based contrast within the fertility workup, taking into account both its diagnostic potential as well as its therapeutic effect. A cost-effectiveness analysis of the long term outcomes of the H2Oil study showed an increase in the cumulative pregnancy rate when oil-based contrast was used, compared to when water-based contrast was used (80,0 versus 75,0%) [36]. The higher price of the oil-based contrast was compensated by a decrease in the need for ART to achieve these pregnancies in the group receiving oil-based contrast, resulting in comparable overall costs. The study concluded that tubal flushing with oil-based contrast was therefore cost-effective in comparison to water-based contrast in women with unexplained subfertility [36]. In the proposed study, a cost-effectiveness analysis will be performed incorporating medical consumption, absence from (paid) work and loss of productivity due to health problems [24].

The mechanism of action of oil-based contrast leading to a fertility enhancing effect is not fully elucidated. Different hypotheses place the point of action in the Fallopian tube [37, 38], the endometrium [39], and the peritoneum [40, 41]. A post-hoc analysis of the H2Oil-study showed that in the group of women with higher pain scores, the ongoing pregnancy rate was higher in women that had received oil-based contrast during HSG when compared to women that had received water-based contrast [42]. These result support the first hypothesis that when using oil-based contrast medium, the pain was caused by an increase in intrauterine pressure prior to dislodgment of pregnancy-hindering debris from the proximal part of otherwise anatomically normal Fallopian tubes [42]. Previous research associated pre-procedural anxiety to a higher experienced pain level during medical procedures [19, 43]. To further investigate the relationship between discomfort or pain during HSG and ongoing pregnancies in the current study, the APAIS questionnaire will be used to score pre-procedural anxiety as a confounder for experienced pain.

Despite reassuring recent research on the prevalence of complications after an HSG, fear of complications is still a reason for some clinicians to withhold use of oil-based contrast [11, 18]. A recent review, without publication date or language restrictions, showed that the incidence of intravasation of contrast in the venous or lymphatic system is higher during tubal flushing with oil-based contrast in comparison to water-based contrast (2.8% and 1.8% respectively, odds ratio 5.05, 95% CI 2.27 to 11.22) in the included RCTs [18]. However, when including only studies that used fluoroscopy screening during HSG, no serious consequences of intravasation were identified. Pelvic infection after HSG is another well-known complication, the previously mentioned review described that in studies published in or after 1960, as antibiotics were not routinely used before, the frequency of infection was 0.55% after HSG with oil-based contrast and 0.35% after HSG with water-based contrast [18]. The proposed study aims to provide information on the complication rate in a population of women that have a higher risk for tubal pathology (because of a previous infection, pelvic surgery or endometriosis). Complications will be reported systematically.

This multicentre RCT will establish whether, for women that are 39 years or older, women with ovulation disorders, and women who have a high risk for tubal pathology, there is a fertility enhancing effect of tubal flushing at HSG with oil-based contrast during fertility work-up and if this is cost-effective.

## Supplementary Information

**Additional file 1. Table S1**: Description of data: WHO Trial registration data set

**Additional file 2**. List of currently participating centres of H2Oil2 as per March 1st 2022 and their local head investigators

## Data Availability

Data and materials for the main research will be published along with publication of results of this study. Requests for study data once the study is completed can be directed to KR, email address k.rosielle@amsterdamumc.nl or VM, email address mijatovic@amsterdamumc.nl.

## Abbreviations

AE: Adverse event
APAIS: Amsterdam Preoperative Anxiety and Information Scale
ART: Assisted Reproductive Technology
CI: Confidence interval
HSG: Hysterosalpingography
iPCQ: IMTA Productivity Cost Questionnaire
IRB: Institutional Review Board
ITT: Intention to treat
IUI: Intra uterine insemination
IVF: In vitro fertilization
NICE: National Institute for Healthcare and Excellence
PCOS: Polycystic ovary syndrome
RR: Relative risk
SAE: Severe adverse event
VAS: Visual Analogue Scale

## References

[1] Mascarenhas MN, Flaxman SR, Boerma T, Vanderpoel S, Stevens GA (2012). National, regional, and global trends in infertility prevalence since 1990: a systematic analysis of 277 health surveys. PLoS Med.

[2] Zegers-Hochschild F, Adamson GD, Dyer S, Racowsky C, de Mouzon J, Sokol R (2017). The international glossary on infertility and fertility care, 2017. Hum Reprod.

[3] ACOG. Infertility workup for the women’s health specialist. Obstet Gynecol. 2019;133:377–84. 10.1097/AOG.0000000000003271 31135764

[4] NICE. Fertility assessment and treatment for people with fertility problems. 2013 01-02-2013. Contract No.: CG 156 2013.

[5] Mills M, Rindfuss RR, McDonald P, te Velde E, Reproduction E, Society Task F. Why do people postpone parenthood? Reasons and social policy incentives. Hum Reprod Update. 2011;17(6):848–60. 10.1093/humupd/dmr026 PMC3529638 21652599

[6] Hoenderboom BM, van Benthem BHB, van Bergen J, Dukers-Muijrers N, Gotz HM, Hoebe C (2019). Relation between *Chlamydia trachomatis* infection and pelvic inflammatory disease, ectopic pregnancy and tubal factor infertility in a Dutch cohort of women previously tested for chlamydia in a chlamydia screening trial. Sex Transm Infect.

[7] Trimbos-Kemper T, Trimbos B, van Hall E (1982). Etiological factors in tubal infertility. Fertil Steril.

[8] Taylor E, Gomel V (2008). The uterus and fertility. Fertil Steril.

[9] Practice Committee of the American Society for Reproductive M. Effectiveness and treatment for unexplained infertility. Fertil Steril. 2006;86(5 Suppl 1):S111–4. 10.1016/j.fertnstert.2006.07.1475 17055802

[10] Bhattacharya S, Porter M, Amalraj E, Templeton A, Hamilton M, Lee AJ (2009). The epidemiology of infertility in the North East of Scotland. Hum Reprod.

[11] Roest I, van Welie N, Mijatovic V, Dreyer K, Bongers M, Koks C (2020). Complications after hysterosalpingography with oil- or water-based contrast: results of a nationwide survey. Hum Reprod Open.

[12] Wang R, van Welie N, van Rijswijk J, Johnson NP, Norman RJ, Dreyer K (2019). Effectiveness on fertility outcome of tubal flushing with different contrast media: systematic review and network meta-analysis. Ultrasound Obstet Gynecol.

[13] Fang F, Bai Y, Zhang Y, Faramand A (2018). Oil-based versus water-based contrast for hysterosalpingography in infertile women: a systematic review and meta-analysis of randomized controlled trials. Fertil Steril.

[14] Wang R, Watson A, Johnson N, Cheung K, Fitzgerald C, Mol BWJ (2020). Tubal flushing for subfertility. Cochrane Database Syst Rev.

[15] Dreyer K, van Rijswijk J, Mijatovic V, Goddijn M, Verhoeve HR, van Rooij IAJ (2017). Oil-based or water-based contrast for hysterosalpingography in infertile women. N Engl J Med.

[16] van Rijswijk J, van Welie N, Dreyer K, Pham CT, Verhoeve HR, Hoek A (2020). Tubal flushing with oil-based or water-based contrast at hysterosalpingography for infertility: long-term reproductive outcomes of a randomized trial. Fertil Steril.

[17] Lindequist S, Rasmussen F, Larsen C (1994). Use of iotrolan versus ethiodized poppy-seed oil in hysterosalpingography. Radiology.

[18] Roest I, Rosielle K, van Welie N, Dreyer K, Bongers M, Mijatovic V (2021). Safety of oil-based contrast medium for hysterosalpingography: a systematic review. Reprod Biomed Online.

[19] Moerman N, van Dam FSAM, Muller MJ, Oosting H (1994). The Amsterdam Preoperative Anxiety and Information Scale (APAIS). Anesth Analg.

[20] Dutch Society of Obstetrics and Gynaecology. Guideline-Anovulation and wish to conceive. 2004. https://www.nvog.nl/wp-content/uploads/2017/12/Anovulatie-en-kinderwens-2.0-12-11-2004.pdf.

[21] Dutch Society of Obstetrics and Gynaecology. Guideline-Basic fertility work-up. 2015. https://richtlijnendatabase.nl/richtlijn/orienterend_fertiliteitsonderzoek_ofo/startpagina_orienterend_fertilitietsonderzoek.html.

[22] Dutch Society for Obstetrics and Gynaecology. Guideline-Unexplained infertility. 2020. https://richtlijnendatabase.nl/richtlijn/onverklaarde_subfertiliteit/startpagina_-_onverklaarde_subfertiliteit.html.

[23] Hunault CC, Laven JS, van Rooij IA, Eijkemans MJ, te Velde ER, Habbema JD (2005). Prospective validation of two models predicting pregnancy leading to live birth among untreated subfertile couples. Hum Reprod.

[24] Manual iMTA Productivity Cost Questionnaire (iPCQ). 2013. www.imta.nl. 10.1016/j.jval.2014.08.1791 27201788

[25] Kanters TA, Bouwmans CAM, van der Linden N, Tan SS, Hakkaart-van Roijen L. Update of the Dutch manual for costing studies in health care. PLoS ONE. 2017;12(11):e0187477. 10.1371/journal.pone.0187477 PMC5679627 29121647

[26] The guidlines manual: Assessing cost effectiveness. 2012. https://www.nice.org.uk/article/pmg6/chapter/7-assessing-cost-effectiveness#economic-evidence-and-guideline-recommendations.

[27] International Conference on Harmonisation of technical requirements for registration of pharmaceuticals for human use. Good Clinical Practice (GCP). 2016. 10.1111/j.1365-2125.1994.tb05705.x PMC1364893 8054244

[28] WHO Trial Registration Data Set. Version 1.3.1. https://www.who.int/clinical-trials-registry-platform/network/who-data-set.

[29] Fertility treatment 2019: trends and figures. 2021 UK statistics for IVF and DI treatment, storage and donation. https://www.hfea.gov.uk/about-us/publications/research-and-data/fertility-treatment-2019-trends-and-figures/.

[30] Preliminary National Summary Report 2019. 2022. https://www.sartcorsonline.com/rptCSR_PublicMultYear.aspx?ClinicPKID=0.

[31] DeCherney AH, Kort H, Barney JB, DeVore GR (1980). Increased pregnancy rate with oil-soluble hysterosalpingography dye. Fertil Steril.

[32] Alper MM, Garner PR, Spence JEH, Quarrington AM (1986). Pregnancy rates after hysterosalpingography with oil- and water-soluble contrast media. Obstet Gynecol.

[33] Schwabe MG, Shapiro SS, Haning RV (1983). Hysterosalpingography with oil contrast medium enhances fertility in patients with infertility of unknown etiology. Fertil Steril.

[34] Steiner AZ, Meyere WR, Clark RL, Hartmann KE (2003). Oil-soluble contrast during hysterosalpingography in women with proven tubal patency. Obstet Gynecol.

[35] Dutch Society of Obstetrics and Gynaecology. National guideline infertility. 2010. https://www.nvog.nl/wp-content/uploads/2018/02/Subfertiliteit-landelijke-netwerkrichtlijn-1.0-20-05-2011.pdf.

[36] van Welie N, Pham CT, van Rijswijk J, Dreyer K, Verhoeve HR, Hoek A (2021). The long-term costs and effects of tubal flushing with oil-based versus water-based contrast during hysterosalpingography. Reprod Biomed Online.

[37] Kerin JF, Williams DB, San Roman GA, Pearlstone AC, Grundfest WS, Surrey ES (1992). Falloposcopic classification and treatment of fallopian tube lumen disease. Fertil Steril.

[38] Roest I (2022). What is the fertility-enhancing effect of tubal flushing? A hypothesis article. Int J Obstet Gynaecol..

[39] Johnson NP, Bhattu S, Wagner A, Blake DA, Chamley LW (2005). Lipiodol alters murine uterine dendritic cell populations: a potential mechanism for the fertility-enhancing effect of lipiodol. Fertil Steril.

[40] Johnson JV, Montoya IA, Olive DL (1992). Ethiodol oil contrast medium inhibits macrophage phagocytosis and adherence by altering membrane electronegativity and microviscosity. Fertil Steril.

[41] Mikulska D, Kurzawa R, Rozewicka L. Morphology of in vitro sperm phagocytosis by rat peritoneal macrophages under influence of oily contrast medium (Lipiodol). Acta Eur Fert. 1994. 7900503

[42] van Welie N, Dreyer K, van Rijswijk J, Verhoeve HR, Goddijn M, Nap AW (2019). Treatment effect of oil-based contrast is related to experienced pain at HSG: a post-hoc analysis of the randomised H2Oil study. Hum Reprod.

[43] Sorrentino F, Petito A, Angioni S, D'Antonio F, Severo M, Solazzo MC (2021). Impact of anxiety levels on the perception of pain in patients undergoing office hysteroscopy. Arch Gynecol Obstet.

