# Supplementary material for: Oil-based versus water-based contrast media for hysterosalpingography in infertile women of advanced age, with ovulation disorders or a high risk for tubal pathology: study protocol of a randomized controlled trial (H2Oil2 study)
Source: BMC Womens Health. 2022 Apr 18;22:123. doi: 10.1186/s12905-022-01707-z (PMC9016997; doi:10.1186/s12905-022-01707-z)
Supplement: Supplementary file 1 — Additional file 1. Table S1: Description of data: WHO Trial registration data set [file 12905_2022_1707_MOESM1_ESM.docx]

Supplement 1, Table S1

| Data category | Information |
| --- | --- |
| Primary registry and trial identifying number | Trialregister.nl , NL7925 |
| Date of registration in primary registry | August 1^st^ 2019 |
| Secondary identifying numbers | EudraCT 2018-004192-12  Protocol number 2005746, version 1.9 21-10-2021 |
| Source of monetary of material support | ZonMw, the Netherlands  Guerbet |
| Primary sponsor | Stichting VUmc |
| Secondary sponsor | N/A |
| Contact for public queries | KR, VM  k.rosielle@amsterdamumc.nl; +3120 444 4567  De Boelelaan 1117, 1081 HV Amsterdam, the Netherlands |
| Contact for scientific queries | Prof. dr. V. Mijatovic, mijatovic@amsterdamumc.nl; +3120 444 4567  De Boelelaan 1117 ,1081 HV Amsterdam, the Netherlands  Study team  H2Olie2@amsterdamumc.nl; +3120 444 4567  De Boelelaan 1117, 1081 HV Amsterdam, the Netherlands |
| Public title | Oil-based versus water-based contrast media for hysterosalpingography in infertile women of advanced age, with ovulation disorders or a high risk for tubal pathology: study protocol of a randomized controlled trial |
| Scientific title | Oil-based versus water-based contrast media for hysterosalpingography in infertile women of advanced age, with ovulation disorders or a high risk for tubal pathology: study protocol of a randomized controlled trial (H2Oil2) |
| Countries of recruitment | The Netherlands, the United Kingdom |
| Health condition(s) or problem(s) studied | Infertility |
| Intervention | Intervention group: HSG with oil-based contrast (Lipiodol Ultra Fluide, Guerbet, Villepinte, France)  Control group: HSG with water-based contrast (iodixanol, Visipaque®, General Electric Healthcare, Buc, France) |
| Key inclusion and exclusion criteria | Inclusion criteria:   - Women ≥18 years of age - Indication for HSG - One of the following: - ≥ 39 years of age and/or - Ovulation disorder (≤8 ovulations / year) - High risk for tubal pathology based on history   Exclusion criteria:   - Endocrine disorder known to decrease pregnancy chances - Iodine allergy - Partner with severe male infertility |
| Study type | Interventional  Allocation: randomized intervention model, not blinded, parallel  Randomization: online using a permuted block design with blocks size 4, 6 and 8, stratified for inclusion site  Primary purpose: treatment  Phase: post market phase |
| Date of first enrollment | August 20^th^ 2019 |
| Sample size | Number of participants planned: 930  Current inclusion number: 266 |
| Recruitment status | Recruiting |
| Primary outcome(s) | Ongoing pregnancy leading to live birth, with first day of last period preceeding pregnancy within 6 months of randomization |
| Key secondary outcome(s) | Other pregnancy outcomes within 6 months of randomization: clinical pregnancy (ultrasound confirmed intrauterine gestational sac), ongoing pregnancy (positive fetal heartbeat on ultrasound examination after 12 weeks of gestation), miscarriage (loss clinical or ongoing pregnancy or diagnosis of a pregnancy without positive fetal heartbeat before 12 weeks gestation), ectopic pregnancy (ultrasound or surgically confirmed extra uterine pregnancy). Pregnancy complications, complications of HSG such as intravasation, infection and hypo- or hyperthyroidism, and a cost-effectiveness analysis will also be part of the secondary outcomes. Procedural pain measured using a VAS-ruler within 10 minutes of the procedure |
| Ethics review | Status: approved  Date of approval: July 22^nd^ 2019  Ethics committee: Medisch Ethische Toetsingscommissie, +3120 444 5585, metc@vumc.nl |
| Completion data | N/A |
| Summary results | N/A |
| IPD sharing statement | The completed study database will be available on an online repository for further research upon reasonable request after an embargo period. |
