# Supplementary material for: Oil-based versus water-based contrast media for hysterosalpingography in infertile women of advanced age, with ovulation disorders or a high risk for tubal pathology: study protocol of a randomized controlled trial (H2Oil2 study)
Source: BMC Womens Health. 2022 Apr 18;22:123. doi: 10.1186/s12905-022-01707-z (PMC9016997; doi:10.1186/s12905-022-01707-z)
Supplement: Supplementary file 2 — Additional file 2. List of currently participating centres of H2Oil2 as per March 1st 2022 and their local head investigators [file 12905_2022_1707_MOESM2_ESM.docx]

Supplement 2: list of currently participating centers H2Oil2 (March 1^st^ 2022)

The Netherlands:

Amsterdam UMC location Vrije Universiteit, Amsterdam, PI Prof. dr. V. Mijatovic

Amstelland Ziekenhuis, Amstelveen, PI Drs. A. Mozes

Reinier de Graaf Groep, Delft, PI Dr. E.J.P. van Santbrink

Elkerliek Ziekenhuis, Helmond, PI dr. J. Penninx

Zaans Medisch Centrum, Zaandam, PI drs. A.B. Hooker

Catharina Ziekenhuis Eindhoven, PI dr. A.G. Huppelschoten

United Kingdom:

Imperial College NHS Healthcare Trust, London, PI prof. dr. A. Hemingway
